# Small Molecule-Photoactive Yellow Protein Labeling Technology in Live Cell Imaging

**DOI:** 10.3390/molecules21091163

**Published:** 2016-08-31

**Authors:** Feng Gao, Tang Gao, Kechao Zhou, Wenbin Zeng

**Affiliations:** 1 Powder Metallurgy Research Institute of Central South University, Changsha 410013, China; gf0731@163.com; 2 The Third Xiangya Hospital, Central South University, Changsha 410013, China; 3 School of Pharmaceutical Sciences, Central South University, Changsha 410013, China; tanggao1990@hotmail.com

**Keywords:** small molecule, photo active yellow protein, live cell, protein labeling, imaging

## Abstract

Characterization of the chemical environment, movement, trafficking and interactions of proteins in live cells is essential to understanding their functions. Labeling protein with functional molecules is a widely used approach in protein research to elucidate the protein location and functions both in vitro and in live cells or in vivo. A peptide or a protein tag fused to the protein of interest and provides the opportunities for an attachment of small molecule probes or other fluorophore to image the dynamics of protein localization. Here we reviewed the recent development of no-wash small molecular probes for photoactive yellow protein (PYP-tag), by the means of utilizing a quenching mechanism based on the intramolecular interactions, or an environmental-sensitive fluorophore. Several fluorogenic probes have been developed, with fast labeling kinetics and cell permeability. This technology allows quick live-cell imaging of cell-surface and intracellular proteins without a wash-out procedure.

## 1. Introduction

Proteins account for some of the largest and most important molecules in living organism, and function through interaction with various biomolecules. Thus, understanding the structure, distribution, trafficking, and environment of the proteins of interests (POIs) in living cells, will not only shed light on elucidating the mechanism(s) of cellular function, but also give ideas for the development of therapeutic or tool drugs, as well as tremendous other biological applications [1,2,3].

The most invaluable method for bioimaging proteins is to genetically encode green fluorescent protein (FP) or its variants as a fusion to the POI, followed by using microscopy to visualize the POI after expression. FP has been extensively used in protein labeling, not only because of its one domain structure which makes it easy and feasible to fuse into the target protein with no interference and dramatic change to the native protein functionalities, but also owing to its compact structure [4,5,6]. However, FPs have some limitations, such as relatively large size (ca. 27 kDa), unsatisfactory fluorescence intensity and photostability, difficult to engineer FP variants with good emission and absorption spectra, etc. [1,7].

**Figure 1:**
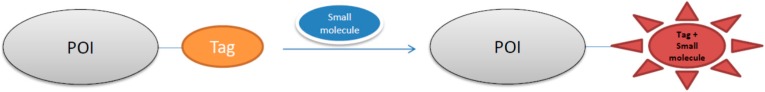
General strategy for labeling protein with small molecules. Protein of interest (gray oval) fusion with peptide or protein tag (yellow oval), which is able to bind to a small molecule (blue oval). Upon binding, the detectable small molecule will give a certain fluorescent signal and protein function is analyzed by fluorescence microscopy or other detection methods.

Another useful protein labeling methodology is to label fusion protein with small molecule probes. In order to label site specific POI in vivo, the most widely used strategy is gene fusion in which a peptide or protein tags into a peptide sequence, protein domain or receptor protein [3,6,8]. First, the small molecule probe comprising a tag binding ligand and a fluorophore or other functional unit is added to a culture of growing cells, and then the probe can go across the cell membrane and specifically bind to the fusion peptide or protein tag to finish the protein labeling (Figure 1) [9]. The essential part of this strategy is to develop a small molecule-based protein-specific probe that doesn’t interfere with the receptor protein. Furthermore, the small molecule should be cell permeable and non-toxic to the cell. An early report described the development of tetracysteine tags [10,11], and later SNAP-tags [12,13], Halo tags [14,15], TMP tags [16], LAPs [17], Oligo-Asp tags [18], coiled-coil tags [19], and BL-tags [20]. Disadvantage of this method is the free small molecule probe needs to be removed by washing the cells or purifying the cell lysate to distinguish the signal of bound and unbound probes, and incomplete washing results in a decrease in the signal-to-noise ratio. Thus, there is great demanding to develop protein labeling probes with no requirement for any washing procedure in live cell imaging. One of the possible solutions to this problem is to develop turn-on fluorescence protein labeling systems, in which the fluorescence of the probe is quenched in a free state and recovered upon binding to the protein tag. The fluorescent probe can largely minimize the background and avoid the conventional washing step. Despite the fact that many protein labeling technologies have been reported, there are quite few protein tag systems that do not require any washing [5,10,11,21,22,23,24,25,26]. In this review, we will summarize the recent progress on using small molecule-photoactive yellow protein (PYP) labeling technology for live cell imaging.

## 2. Photoactive Yellow Protein

Photoactive yellow protein (PYP) is a small, water-soluble, yellow colored acidic protein, which was first found in the purple photosynthetic bacterium *Halorhodospira* *halophile* (Ectotiorhodospirahalophila) [27,28]. PYP can be bleached by light stimulus but the color could recover within 1 s in dark [29]. The first high-resolution structure of PYP was established by crystallography in 1995 [30]. To date, more than 140 PYP genes have been found in a wide variety of bacteria and metabolic niches, and according to their amino acids sequence and physical properties, PYP-like proteins and PYP-related domains could be divided in to seven groups (Table 1) which have different functions [31].

PYP contains 125 amino acids with a molecular weight of ca. 14 k Da (half the size of the green fluorescent protein, GFP), and it id folded into an α/β fold structure with a central six-stranded β sheet and five short α helices [30,32]. This structure belongs to the superfamily of the PerArntSim (PAS) domain, which is found to be widely distributed among humans and bacteria. One of the most important functions of PAS domain protein is working as sensor for stimuli, such as light, voltage and oxygen [33]. However, PAS domains generally exist as part of a multi-domain protein, while PYP only functions solely on the PAS domain, suggesting PYP is a structural protype for PAS domain proteins [33].

**Figure 2:**
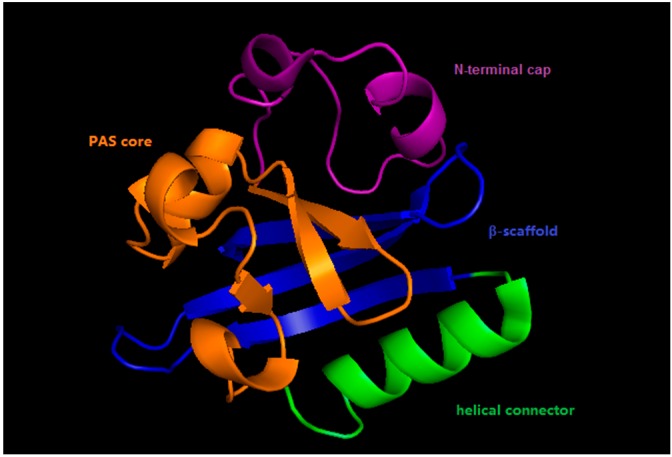
Photoactive yellow protein structure (PDB: 2phy). The overall structure of PYP is divided into four segments: N-terminal cap (purple), contains residues form 1 to 28; PAS core (orange), where high sequence homology has been found among various PAS-containing molecules, contains residues from 29 to 69; Helical connector (green), spans from residue 70 to 87; β-scaffold (blue), spans from residues from 88 to 125, contains the last three strands of PYP.

Like the PAS domain protein, the structure of PYP could be divided into four segments: β-scaffold, helical connector, PAS core and N-terminal cap (Figure 2) [34]. The β-scaffold is composed of long β-strands and the loops that connect them. The helical connector is composed of the C-terminal half of the chromophore binding site and a long α-helix. The PAS core segment is composed of short β-strands, α-helices and the N-terminal half of the chromophore binding site. The N-terminal cap is composed of short α-helices and loops, with the chromophore located on the other side of the β sheet [33].

As a photoactive protein, PYP consists of a 4-hydroxycinnamic acid (also called *p*-coumaric acid, pCA) which covalently bound to Cys69 through transthioesterification. It has been proven that the covalent linkage is crucial for the PYP photocycle [35]. The *p*-coumaroyl chromophore of PYP has one ionizable oxygen atom (phenolic oxygen, O4′), and one isomerizable double bond which undergoes *trans-cis* isomerization during the photocycle: in the dark state, O4′ in the chromophore is deprotonated and stabilized by a hydrogen-bonding network with Tyr42, Glu46, Thr50 and the chromophore [30], together with the double bond is in *trans* form. Upon absorption of photons, the double bond is isomerized to the *cis* form, followed by protonation of the chromophore by its hydrogen bonding partner Glu46 [36,37]. The absorption maximum of PYP from Hr. halophile is located at 446 nm, which gives it its bright yellow color [29].

**Scheme 1:**
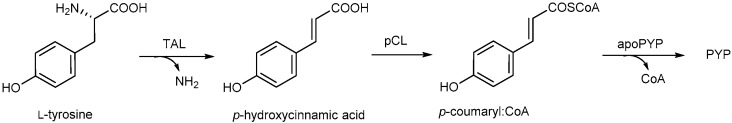
Crucial steps for biosynthesis of *p*-coumaryl: CoA. TAL: tyrosine ammonia lyase; pCl: *p*-coumaryl: coenzyme A ligase.

It was shown that *p*-coumaric acid is generated from tyrosine by tyrosine-ammonia lyase, followed by attachment to the thiol group of coenzyme A by *p*-hydroxycinnamic acid ligase (Scheme 1) [38,39]. The generation of thioester bond between the *p*-coumaric acid and cysteine is difficult, but this was solved by coupling *p*-coumaric anhydride or *p*-coumaric thiophenyl ester with the thiol group of the cysteine residue to reconstitute PYP [40,41]. By using this reconstitution strategy, wild type PYP and its analogs could be produced using an *Escherichia coli* over-expression system, which facilitates much higher yield compared with conventional extraction and purification approach from the *Hr. halophile* culture medium [35]. In addition to the natural cofactor, it is reported that PYP also binds to other fluorescent compounds, such as 7-hydroxycoumarin-3-carboxylic acid thioester derivatives [35].

## 3. Small Molecule-Photoactive Yellow Protein Labeling Technology in Live-Cell Imaging

### 3.1. Development of Turn-On Fluorescent Protein Labeling System Based on Photoactive Yellow Protein

**Figure 3:**
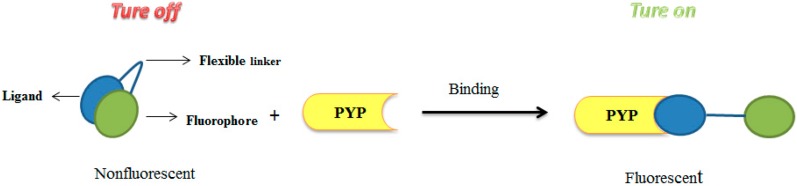
Principle of fluorogenic labeling system based on PYP.

**Scheme 2:**
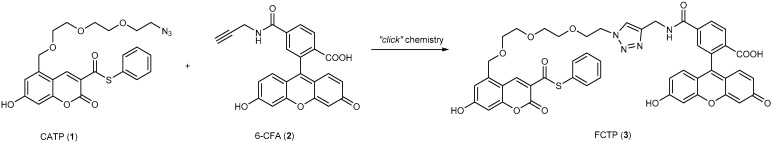
Structures of fluorescent probes CATP and FCTP.

PYP is a small size protein with interesting functions, and exclusively existing in bacteria. It is expected that PYP expressed in animal cells will not led to any cross-reactions with endogenous factors. Kikuchi and coworkers smartly designed a turn on protein labeling system which connected coumarin derivatives with another fluorescein by a flexible linker based on photoactive yellow protein (Figure 3) [42]. The protein labeling system have no fluorescence because of the intramolecular association between the fluorophores, but their fluorescence intensity will be restored upon the dissociation between coumarin derivative and its linked fluorescein [43]. In the absence of PYP, the probe is not fluorescent because of the intramolecular association. Once the probe binds to the PYP, the association between the coumarin and fluorescein is interfered, which results in the dissociation of two units and increased fluorescence intensity. Probe FCTP (**3**) was prepared by conjugating coumarin derivative CATP (**1**) with fluorescein 6-CFA (**2**) via click chemistry (Scheme 2). The ethylene glycol linker introduced into CATP at the 5-position of coumarin is to reduce the steric hindrance [44]. Binding properties were characterized by incubating the probes with recombinant PYP purified from *E. coli* and analyzed by SDS-PAGE analyses.

In mixtures of the probes CATP and FCTP with PYP the fluorescent bands was detected in the gel, which indicated that both CATP and FCTP bind to PYP. This was also confirmed by MALDI-TOF MS experiments, whilst CATP and FCTP covalently bind to PYP through transthioesterification by replace the thiophenyl ester with the thioester of the cysteine from the protein. Binding specificity of the probes was also investigated by labeling the purified PYP in the lysate prepared from HEK293T cells, and it was found out that PYP was specifically labeled by CATP or FCTP. Furthermore, the presence of high concentrations of glutathione which contains free thiols did not affect the labeling reactions [45]. It was interesting to find out that after labeling the PYP with CATP, the azido group in CATP would allow additional labeling in cell lysates by click chemistry, this greatly expanded the usage of this technology.

The fluorogenic properties of FCTP were examined by measuring fluorescence spectroscopy. The fluorescence intensity of FCTP was very weak in the absence of PYP, due to the association between the coumarin and fluorescein in the probe. On the other hand, the binding of PYP and the probe led to a 20-fold increase of fluorescenve intensity, indicated binding of the PYP with the probe could cause the dissociation between the coumarin and the fluorescein. CATP and FCTP have different binding kinetics, whereas CATP completed the binding reaction with PYP in 2 h, FCTP required more than 24 h, suggesting that the intramolecular interaction in FCTP affects its binding kinetics. CATP and FCTP are able to label live cells. HEK293T cells expressing PYP-PDGFRtm (the fusion protein of PYP and a transmembrane domain of platelet-derived growth factor receptor) could be labeled by CATP and FCTP, while cells without PYP-PDGFRtm could not be labeled. CATP was cell-permeable, and could label PYP in the living cells expressing maltose binding protein-fused PYP (MBP-PYP) in the cytosol.

### 3.2. Development of Protein-Labeling Probes for No-Wash Live Cell Imaging

FCTP as a probe for protein labeling provides another option for live cell imaging. However, the labeling reaction of the probes to PYP was slow (it requires for more than 24 h to complete the labeling reaction), which results in an enhanced signal-to noise ratio. Thus, development of flurogenic probes with fast kinetics is of great interest for no-wash live cell imaging.

**Figure 4:**
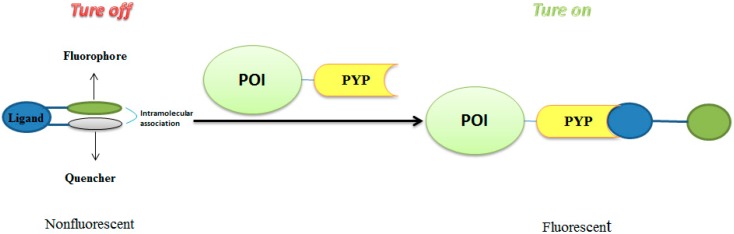
Principle of fluorogenic probes with a turn on/off quencher.

**Figure 5:**
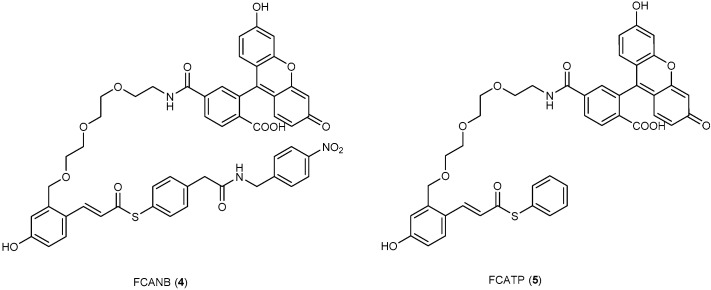
Chemical structures of FCATP and FCANB.

Design of the new probe FCANB (**4**) was based on the following hypothesis (Figure 4): (a) the coumarin moiety in FCTP is replaced by a cinnamic acid thioester, which could bind to PYP and is expected to reduce the interaction between the ligand and the fluorophore; (b) introduction of a nitrobenzene moiety which is supposed to associate with the fluorophore, thereby quench its fluorescence; (c) the association between nitrobenzene and fluorophore inhibits the undesired interactions between the cinnamic acid thioester ligand and the fluorophore moiety, which is probably the reason why FCTP has slow kinetics; (d) on binding of the ligand to PYP, the thiophenyl leaving group which connects to nitrobenzene will be cleaved off, thus restoring the probe’s fluorescence. In order to investigate the effect of the nitrobenzene moiety, FCATP without the nitrobenzene motif was also designed and synthesized (Figure 5) [46]. The binding abilities of FCANB and FCATP were examined with SDS-PAGE. It was confirmed that both probes could covalently bind to PYP. The binding is specific, which was proved by adding probes to the cell lysate with or without PYP-tag, and later characterized by SDS-PAGE experiments. Both FCATP and FCANB exhibited slight fluorescence in the absence of PYP, but binding of FCATP and FCANB to PYP tag led to 9.3- and 15-fold enhancement of the fluorescence, respectively. It was interesting to find out that even without a quenching group, FCATP exhibited slight fluorescence. Neither FCATP nor FCANB displayed a time dependent alteration of the fluorescence intensity in the absence of PYP, which demonstrates that both FCATP and FCANB could be used as fluorogenic probes for labeling PYP tags. The maximum absorption wavelength of free FCATP and FCANB was shifted by 7 nm in comparison to that of the fluorescein derivative without a ligand or nitrobenzene moiety. It strongly suggested that an intramolecular association between the fluorophore and nitrobenzene or ligand existed [42,43,47,48]. Kinetic studies were performed to determine the *t*_1/2_ (time required for 50% labeling). FCANB binds to PYP tag rapidly with the shortest *t*_1/2_ (ca. 15 min), followed by FCATP (*t*_1/2_, ca. 78 min) and FCTP (*t*_1/2_ > 470 min). Second-order rate constant consistent with the *t*_1/2_ values, the *k*_2_ values of FCATP and FCANB are 10-fold and 110-fold higher than that of FCTP, respectively. All the kinetic studies suggested that introduction of a 4-hydroxycinnamic acid ligand instead of a coumarin ligand leads to a fast protein labeling. It is more likely that the change of the intermolecular association between the fluorophore and the ligand can affect the protein labeling kinetics for FCANB and FCATP. The different kinetics between FCANB and FCATP, indicated that the fluorophore favorably binds to nitrobenzene other than the ligand, thereby reducing the steric hindrance around the ligand moiety.

FCANB and FCATP could be used in live cell imaging. Human embryonic kidney (HEK293T) cells expressing epidermal growth factor receptor (EGFR) fusion with PYP tag could be labeled by either FCANB or FCATP after 30 min of incubation, and the labeling was specific to PYP tags. Cells expressing EGFR without PYP will not be labeled. A washing step after the labeling reaction was not essential for the imaging, while the fluorescence of the free probe was not detectable in the media or in other parts of the cell.

### 3.3. Development of Flurogenic Probes for Imaging of Intracellular Proteins in Living Cells

**Figure 6:**
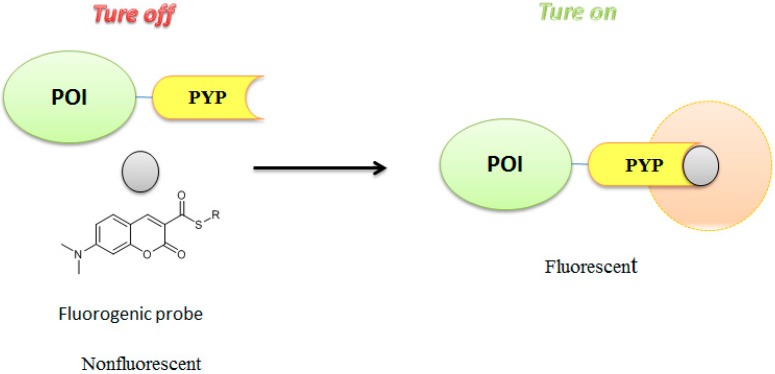
Principle of the labeling system based on PYP-tag and environment-sensitive fluorogenic probe.

FCTP can be used as a probe to label proteins, but it has slow labeling kinetics which requires more than 24 h for the full PYP-tag labeling, thus a washing step was necessary to improve the signal-noise ratio and this limits the applications of FCTP as a protein labeling probe. FCANB was found to have fast labeling kinetics and enable to imaging cell surface proteins without a washing step. It is found that FCANB could not cross the cell membrane. It is necessary to develop cell permeable fluorogenic probes for labeling PYP-tagged proteins with fast incubation time in living cells. 7-Dimethylamino-coumarin derivatives are environment-sensitive fluorophores, which have weak fluorescence in polar solvents, but become fluorescent in less polar environments (Figure 6) [49]. Besides, 7-dimethylaminocoumarin thioester is structurally similar to cinnamic acid and coumarin, thus it was expected to be a probe for binding PYP-tag through transthioerstification [50]. It was envisioned that a PYP-tag probe with this structure would scarcely fluoresce in aqueous buffer, but emit strong fluorescence after the probe binds to the PYP-tag, which was believe to be the probe was brought into a low-polar environment after binding.

**Figure 7:**
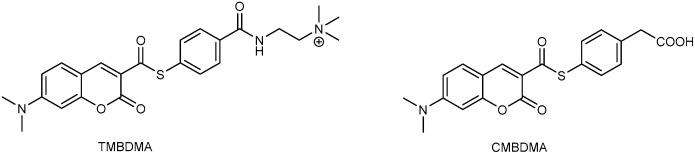
Chemical structures of TMBDMA and CMBDMA.

TMBDMA and CMBDMA (Figure 7) were designed and synthesized as probes based on the hypothesis that the trimethylamine or carboxylic acid moieties introduced into the probes to increase the solubility for both compounds [51]. In vivo experiments showed that both TMBDMA and CMBDMA covalently bind to PYP-tag, as confirmed by SDS-PAGE. The binding was specific to PYP-tag, other thiol compounds, like glutathione [45], could not interfere the binding even in a high concentration (up to 10 mM). Upon protein labeling, the fluorescence intensity at 487 nm was increased by 22-fold and 16-fold for TMBDMA and CMBDMA, respectively. There was no substantial change of the fluorescence intensity in the absence of PYP-tag in 3 h period, demonstrated that TMBDMA and CMBDMA are fluorogenic probes for labeling PYP-tag. Kinetic study showed TMBDMA had the *t*_1/2_ of 1.1 min with the protein and probe concentration at 5 µM, while CMBDMA had the *t*_1/2_ of 13 min. Second-order rate constant for reaction between PYP-tag and probes showed TMBDMA had remarkable value (*k*_2_ = 3950 M-1s-1) which was 32-fold higher than FCANB (*k*_2_ = 125 M-1s-1), with CMBDMA (*k*_2_ = 126 M-1s-1) being comparable to that of FCANB. There are two possible reasons for this difference: one possible reason is that electrostatic interaction between two probes is different, PYP-tag is anionic under the physiological conditions, and is more favorable to interact with cationic TMBDMA other than anionic CMBDMA. The other reason is that TMBDMA contains a better leaving group than CMBDMA. When Cys69 from the PYP-tag attacks the thioester of the probe, the leaving ability of the thiophenyl compound is correlated with the labeling kinetics. Compared with CMBDMA, TMBDMA has a better leaving group which has lower p*K*_a_ owing to the direct connection of a strong electron-withdrawing group to the thiophenol.

Both TMBDMA and CMBDMA could be used as probes for live-cell imaging of cell surface protein, which was prepared by fusing PYP-tag to EGFR. The labeling was fast (30 min), specific to PYP-tag, and no washing step was needed. Even if a washing step was carried out after the labeling reaction, the imaging displayed fluorescence localization, which was essentially similar to the image obtained by the no-wash labeling, indicated that the probes could specifically label PYP-tagged proteins on the cell surface without a washing step.

TMBDMA and CMBDMA () could label intracellular proteins. Maltose-binding proteins fusion with PYP-tag (MBP-PYP), which was mainly expressed in the cytosol, could be specifically labeled immediately after incubating the cells with TMBDMA for 30 min. Cell expressing MBP without PYP-tag didn’t exhibit fluorescence. The similar pattern was also found when labeling PYP-tagged nuclear localization signals (PYP-NLS). CMBDMA also imaged localization of MBP-PYP and PYP-NLS, which was identical to that of TMBDMA. In all experiments, data from no-wash imaging were indistinguishable from those with a washing step, suggested both TMBDMA and CMBDMA are cell permeable, and could specifically label intracellular PYP-tag fusion proteins without a washing step. Time-lapse imaging experiments showed addition of TMBDMA to cells expressing PYP-NLS, fluorescence started to appear in 2 min and was saturated within 6 min, while CMBDMA needs 30 min to saturate the fluorescence intensity, which is consistent with in vitro experiments where CMBDMA requires longer incubation time compared with TMBDMA. TMBDMA and CMBDMA could also be used as probes to imaging 5-methylctosine (5-mC) by labeling protein fused of PYP-tag and MBD1 (1-112) (PYP-MBD) [51], and localized that PYP-MBD was predominantly in heterochromatin in the cell.

### 3.4. Turn-On Fluorescent Protein Labeling System Based on Mutant PYP-tag

**Figure 8:**
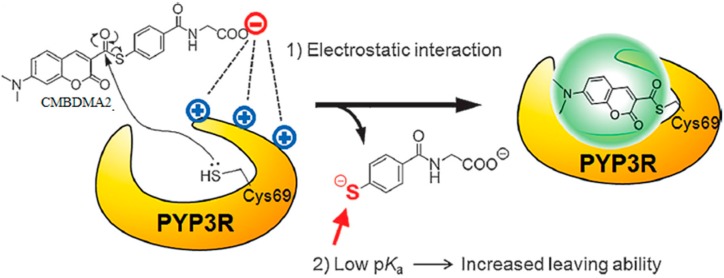
PYP-tag mutant PYP3R and its fluorogenic probeCMBDMA2 based on electrostatic interactions and the p*K*_a_ value of the leaving group.

The critical factors for the precise spatiotemporal imaging of protein dynamics in living cells are labeling time and fluorescence contrast of the fluorogenic probes. To solve these problems, researchers have taken mutational and chemical approaches to increase the labeling kinetics and fluorescence intensity of fluorogenic PYP-tag probes. Kikuchi and coworkers have successfully designed a PYP-tag–fluorogenic-probe pair by using mutational and chemical approaches based on electrostatic interaction and p*K*_a_ values. They created a PYP-tag mutant, in which the charges on the protein surface were modulated, and designed fluorogenic probe CMBDMA2 (Figure 8). As studies indicated that the charges of PYP-tag contribute to its labeling kinetics, they created a novel mutant with three arginine residues at these amino acid positions, PYP3R. Moeroever, when ligand binds to PYP-tag through transthioesterification with Cys69, a thiophenyl moiety is released from the probe. Early studies suggested that the p*K*_a_ value of the leaving group could affect the labeling kinetics, so they constucted the fluorogenic probe CMBDMA2. Due to the mutations in PYPtag and probe redesign, the labeling reaction was accelerated by a factor of 18 in vitro. Using this system intracellular proteins were detected with only 1 min. The fluorescence intensity of the probe both in vitro and in living cells was enhanced by the mutant tag. Furthermore, they applied this system to the imaging analysis of bromodomains. The labeled mutant tag successfully detected the localization of bromodomains to acetylhistone and the disruption of the bromodomain–acetylhistone interaction by a bromodomain inhibitor [52].

**Figure 9:**
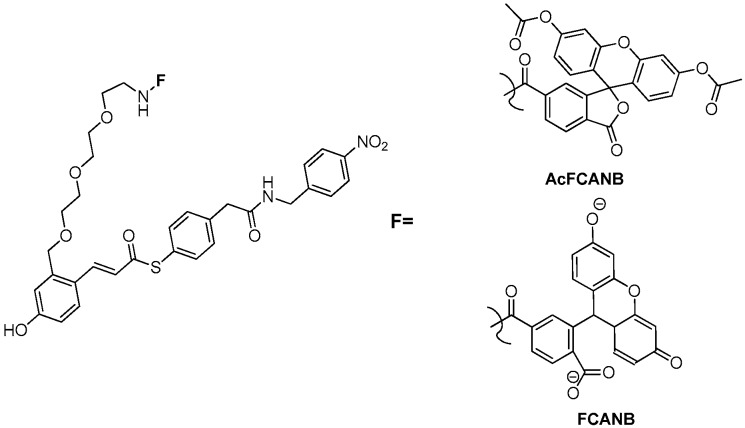
Structures of AcFCANB and FCANB.

Using similar strategies, Kikuchi and coworkers accelerated the labeling reaction by strategic mutations of charged residues on the surface of PYP. They introduced site-selective mutations on the PYP-tag to modulate surface charges and enhance the labeling rate. Furthermore, they successfully developed a simple computational model that quantitatively reproduces the cooperative effect of PYP tag mutations on the kinetics of probe binding. They designed a series of cationic PYP mutants (PYP 4R)and created by point mutation of the acidic amino acids: D53R, D71R, E74R, D97R. To label the intracellular proteins, they constructed a fluorogenic probe, AcFCANB (Figure 9), with neutral charge and membrane permeable. Once incorporated inside the cells, AcFCANB is rapidly digested by cellular innate esterases recovering the anionic FCANB. The electrostatic interactions between the anionic probe and PYP was effectively accelerated the labeling reaction. Results indicated that the AcFCANB and PYP 4R pair system enabled no-wash imaging of intracellular proteins in a desirable time frame (*t*_1/2_ < 30 min), without accumulation or adhesion of the tag protein or the probe to non-targeted organelles. Moreover, in vitro, in silico, and live-cell results were highly consistent, and enabled the physical basis of the improved reaction rate to be clarified [53].

## 4. Conclusions

Collectively, this mini review summarized recent studies on the development of no-wash small molecule probes for using photoactive yellow protein as a tag for imaging proteins. After rational design and synthesis, there are three generations of probes which could be used to imaging PYP-tagged proteins on the cell surface or inside the cell. The labeling reaction is fast and specific. More importantly, no washing step was needed to localize the proteins by using those probes. This technology can be used to clarify various important physiological processes by fluorescence microscopy analysis.

As PYP could be used as a protein tag fusion to a protein of interest, it will further facilitate the utility of this technology by developing small molecule probes with high fluorescent intensity, fast kinetics, ideal cell permeability, easy preparation, etc. In addition, molecular probes with fast kinetics could be used in real time imaging. One other possibility to expand the usage of this technology to modify or optimize the sequence of photoactive yellow protein, to make it more easy to be fused into proteins of interest, thus enabling this protein tag to be more useful to study the diversity of protein localization and functions. The small moleculw probes that have been developed so far are bound to the PYP tag through transthioesterification, however the irreversible thioesterification potentially causes perturbations of the function for the protein, thereby development of PYP variants and small molecular probes with reversible binding, and the small molecular probes could be released from the PYP tag after imaging under mild conditions, will make this technique more attractive. Last but not least, orthogonal imaging of proteins is of high interest, therefore development of the incorporation of different protein tags that compromise with each other into the proteins of interest, followed by applying different molecular probes and imaging methods to selectively study each protein, will shed light on multiple proteins’ localization, and functions research.

## Figures and Tables

**Table 1:** Summary of PYP.

Species	Function/Pathway
*Rs. centenum*	Polyketide synthase
	Cyst formation
	Phototaxis
*Tc. tepidum*	
*Methylomicrobium*	Phototaxis
*Burkholderia*	Gas vesicles 4
*Haliangium*	(Cell buoyancy)
*Curvibacter*	
*Phodobacter*	
*Rp. palustris*	DNA repair (DPL)
*Rt. Salexigens*	Blue light sensor (DGL)
*Desulfonema*	DNA repair (DGL)
*Idiomarina*	Osmotic pressure
*Halorhodospira*	Phototaxis
*Stigmatella*	Stalk formation
	(cyst formation)
*Spirosoma*	
